# A Critical Examination of the Usefulness of Taxonomies for Comparing Cognitive Functions Across Sports

**DOI:** 10.1002/ejsc.70209

**Published:** 2026-06-19

**Authors:** Laura Will, Lisa Musculus, Karen Zentgraf, Hanna de Haan, Dennis Redlich, Markus Raab

**Affiliations:** ^1^ Performance Psychology German Sport University Cologne Institute of Psychology Köln Germany; ^2^ Department 5: Psychology & Sports Sciences Movement and Exercise Science Goethe University Frankfurt Frankfurt am Main Germany

**Keywords:** athletes, cognition, cognitive functions, taxonomy

## Abstract

Taxonomies are widely used in sport science to classify disciplines according to structural, cognitive, and physiological demands, yet their robustness in differentiating athletes' cognitive performance remains uncertain. This study examined whether commonly applied sport classification frameworks—open versus closed skills, strategic–static–interceptive, participant classification, and multidimensional team/precision‐skill/speed–strength—meaningfully represent cognitive differences within junior and senior national squad athletes. A total of 595 national squad athletes from eight sports (artistic gymnastics, rhythmic gymnastics, trampoline, basketball, volleyball, ice hockey, table tennis, modern pentathlon) completed standardized measures of basic (processing speed, attention) and higher (working memory, inhibition, cognitive flexibility) cognitive functions. Using a model comparison approach, we show that the usefulness of taxonomic contrasts is limited because taxonomy‐based models did not improve model fit relative to the null model or the model containing single sport disciplines. For processing speed, single disciplines provided the best fit. Supplementary analyses showed that processing‐speed–based norm scores were above population means and moderately related to attention, but only weakly related to working memory and unrelated to executive control measures. Together, these findings suggest that conclusions about cognitive differences among high‐performance athletes cannot be reliably made using taxonomic classifications as they lack robustness under realistic sampling conditions. The results underscore the importance of sport‐specific and individual‐level approaches when investigating cognitive functioning in high‐performance sport contexts.

## Highlights

- Common sport taxonomies showed limited robustness in differentiating cognitive performance in national squad athletes.
- Broad taxonomic groupings did not yield consistent differences in working memory, inhibition, or cognitive flexibility.
- Findings support sport‐specific and individual‐level approaches to cognitive assessment in high‐performance sport.

## 1 Introduction

While table tennis players who regularly react to high‐speed, fast‐rotation balls with up to 10k revolutions per minute should always excel in processing speed, soccer players who can adapt mid‐game to previously planned tactical positioning should demonstrate superior working memory and cognitive flexibility (e.g., A. Yongtawee et al. 2021; Krenn et al. 2018). Are such strong claims true or an elegant attempt to make things a little less complex? In recent years, sports scientists and cognitive researchers have increasingly relied on taxonomies to classify and systematize sports according to their structural, physiological and cognitive demands (e.g., open vs. closed skill or self‐vs. externally‐paced, see below). Such classifications are intended to provide a structured way to compare athletes' cognitive functions across diverse disciplines. Their development was primarily theoretically motivated, aiming at reflecting underlying cognitive, perceptual, and motor processes across sports (e.g., Singer 2000) and understanding how much they vary by different environmental conditions. They also served methodological purposes like enabling better grouping thereby increasing statistical power and robust comparisons while maintaining conceptual clarity. While they offer a convenient and seemingly systematic means of analysis, especially when grouping due to small samples available is required, they might also oversimplify the complex and context‐dependent realities of athletic performance. This article, therefore, critically examines the usefulness of taxonomies for comparing cognitive functions across sports. It questions whether the reduction of complexity conceals the individuality of cognitive demands across sports to such an extent that these classifications hinder our understanding of how training in different disciplines shapes cognition. Thanks to access to an exceptionally large sample of elite athletes from different sports, we compared results of cognitive diagnostics according to different taxonomic classifications and debate whether an individual lens, that is, a sport‐specific approach that recognizes the complexity and diversity of cognitive demands in athletic contexts, is required to meaningfully discuss differences in cognitive performance.

### 1.1 Sport Taxonomies

In sports, different taxonomies have been developed differing in scope, underlying criteria, and granularity, often leading to divergent groupings and interpretations. While some taxonomies focused on skill complexity and progression at task‐level for therapeutic or instructional use (e.g., Gentile 1972) or conceptions of movement learning (e.g., Newell 1986), others considered entire sport contexts and focus on cognitive demands—which are predominantly relevant for the study at hand. Among those, a foundational classification distinguishes between open‐ and closed‐skill sports, a dichotomy elaborated by Singer (2000) based on earlier work by Knapp (1963). Open‐skill sports are performed in unpredictable, externally driven environments, requiring athletes to continuously adapt to changing stimuli. Examples include soccer, basketball, and tennis. In contrast, closed‐skill sports occur in stable, self‐regulated contexts where movements are largely pre‐planned and consistent, as seen in gymnastics, running, or swimming.

Building on these distinctions, Mann et al. (2007) and Voss et al. (2010) moved beyond the simpler open‐closed skill dichotomy and proposed a tripartite taxonomy which considers the interaction between environmental dynamics (i.e., timing/time pressure), team structure (i.e., number of athletes on the team), and decision‐making: strategic versus interceptive versus static sports. Strategic sports (e.g., basketball, hockey) demand continuous decision‐making in relation to multiple teammates, opponents, and objects, often under conditions of high uncertainty. Interceptive sports (e.g., table tennis, fencing, boxing) emphasize coordination between the body and a rapidly moving implement or opponent, requiring precise timing and sensorimotor integration. Static sports (e.g., swimming, archery) are defined by their stable environmental conditions and predominantly self‐paced execution, minimizing real‐time adaptation. An alternative, related taxonomy categorizes sports according to whether they are self‐paced or externally‐paced, a framework employed in studies, for instance, by C. H. Wang et al. (2013) or Ballester et al. (2019). Self‐paced sports, like golf, track and field, and weightlifting, allow athletes to initiate actions at their own discretion, often involving preparatory routines and internally driven timing. Conversely, externally paced sports—such as football, volleyball, and martial arts—require rapid responses to external cues, placing higher demands on perception, attention, and decision‐making under time pressure.

Expanding beyond cognitive and environmental features, McKay et al. (2021) proposed a seven‐category classification system based on a combination of physiological demands, tactical complexity, and skill requirements. These categories include team sports, endurance or long‐distance sports, middle‐distance or power sports, speed and strength sports, precision or skill‐dependent sports, racquet sports, and combat or weight‐making sports. This framework aims to provide a broader performance‐based taxonomy that acknowledges both physical and technical aspects of athletic practice.

Vona et al. (2024) recently proposed grouping sports into only three categories again—team sports, precision/skill‐dependent sports, and speed/strength sports—based on both motor and energetic demands. Team sports involve complex, dynamic play environments with high requirements for coordination, endurance, and decision‐making. Precision and skill‐dependent sports, such as artistic swimming and figure skating, emphasize fine motor control, spatial awareness, and the execution of choreographed routines. In contrast, speed and strength sports, including alpine skiing and short‐track speed skating, prioritize explosive power and high‐speed maneuvers, often under time‐constrained conditions.

The present study aims at systematically comparing the explanatory value of commonly used sport taxonomies for cognitive performance within a single large sample of national squad athletes. It does not seek to demonstrate that sport taxonomies are either universally valid or fundamentally flawed. Instead, we aim at examining (a) whether cognitive performance is better explained by individual sport disciplines or by broader taxonomic classifications, (b) whether the explanatory value of these classification systems differs across cognitive domains, and (c) whether existing taxonomies capture meaningful variance beyond sport‐specific differences. Once models suggest that single sport discipline models are most plausible to examine cognitive differences, we present further insights to check whether differences in basic and higher cognitive functions appear in the expected direction. Based on prior literature, which we will present below, we expected potential advantages for open‐skill and interceptive sports in attentional performance and for strategic sports in executive control; however, we expected substantial within‐category variability under realistic elite‐athlete sampling constraints (Table 1).

**TABLE 1:** Overview of the results on differences in cognitive functions across different sport taxonomies.

Taxonomy	Basic cognitive functions		Higher cognitive functions		
	Processing speed	Attention	WMC	Inhibition	Flexibility
Open/closed	(±)	✓ (e.g., Zhu et al. 2020; Ren et al. 2025; Giglia et al. 2011)	✓ (e.g., Zhu et al. 2020; Ren et al. 2025)	✓ (e.g., Zhu et al. 2020; C. H. Wang et al. 2013)	✓ (e.g., Gu et al. 2019; Ren et al. 2025)
Strategic/Interceptive/Static	✓ (e.g., Voss et al. 2010; N. Yongtawee and Woo 2021)	(±) (e.g., Krenn et al. 2018; Meng et al. 2019; Rahimi et al. 2022)	✗ (e.g., Krenn et al. 2018)	✓ (e.g., Krenn et al. 2018; Yu et al. 2019; Voss et al. 2010)	✓ (e.g., Meng et al. 2019; strategic athletes; Rahimi et al. 2022)
Participant classification	✗	✓ (e.g., Logan et al. 2022; aerobic/HIIT)	✗	✓ (e.g., Logan et al. 2022; resistance athletes)	✓ (e.g., Logan et al. 2022; aerobic & HIIT)
Team/Skill/Speed–Strength	✓ (e.g., Logan et al. 2022; aerobic athletes)	✓ (e.g., Logan et al. 2022; Vona et al. 2024)	✗ (e.g., Vona et al. 2024)	✗ (e.g., Vona et al. 2024)	✓ (e.g., Vona et al. 2024)

Abbreviations: (±), mixed, task‐dependent, or inconsistent findings; ✓, consistent advantage reported for at least one category within the taxonomy; ✗, no consistent differences reported; HIIT, High‐Intensity Interval Training; WMC, Working Memory Capacity.

#### 1.1.1 Differences in Cognitive Function According to the Different Taxonomies

Let us first review selected empirical evidence on differences in cognitive functions according to taxonomies which differ in their degrees of complexity (i.e., binary vs. tripartite vs. > 5 categories). In line with recent empirical work (Kalén et al. 2021), we differentiate basic versus higher cognitive functions. Basic cognitive functions rely primarily on a single cognitive ability and are relatively stable over the lifespan (Best and Miller 2010; Paz‐Alonso et al. 2014), such as processing speed and attention. Higher cognitive functions involve executive and control processes, coordinating multiple basic functions and are therefore highly effortful, such as core executive functions (i.e., working memory updating, inhibition and interference control, shifting) and decision making, and are more prone to adaptation and change.

##### 1.1.1.1 Open versus Closed‐Skill

Under this taxonomy, open‐skill sports are expected to outperform closed‐skill sports primarily on higher rather than basic cognitive function, because numerous studies suggest that participation in open‐skill sports is associated with superior cognitive performance particularly in executive functions (for an overview see Diamond 2013). In a systematic review, Gu et al. (2019) found that 12 out of 14 observational studies reported cognitive benefits for open‐skill athletes compared to closed‐skill athletes, particularly in inhibitory control and cognitive flexibility, with several intervention studies (i.e., 3 out of 5) further indicating greater gains following open‐skill exercise. Importantly, the pattern of open‐skill activities being associated with superior performance in executive tasks has been reported in both children and adolescents, although findings in younger samples are more heterogeneous and often based on small‐scale interventions. More converging evidence is provided by multiple experimental and cross‐sectional studies showing advantages for open‐skill athletes in higher cognitive functions (e.g., Fleddermann et al. 2023; Heppe and Zentgraf 2019; Memmert 2009; Nougier et al. 1996). Meta‐analytic findings support the pattern: from 21 studies, Zhu et al. (2020) concluded that open‐skill athletes consistently outperformed closed‐skill athletes on tasks assessing working memory, cognitive flexibility, and decision‐making, although they noted that the effect sizes varied across age groups and study designs. Similarly, a recent systematic review by Lai et al. (2024) reported consistent evidence that open‐skill sports are more beneficial than closed‐skill sports for improvements in specific executive‐function components, particularly inhibition, cognitive flexibility, and working memory, while also highlighting substantial heterogeneity in tasks, sport classifications, and study quality. More recently, Ren et al. (2025) confirmed advantages for open‐skill athletes in working memory and cognitive flexibility across different performance levels, while reporting less consistent effects for inhibition. These findings suggest that not all executive components may be equally sensitive to sport type and that cognitive flexibility and working memory may be particularly relevant for dynamic and more unpredictable environmental demands in open sports.

Experimental evidence further supports an effect for specific executive functions. C. H. Wang et al. (2013) compared tennis players (open‐skill), swimmers (closed‐skill), and non‐athletic controls using a stop‐signal task and found significantly shorter stop‐signal reaction times in tennis players, indicative of superior inhibitory control, but swimmers did not differ from non‐athletes. Similar patterns have been observed in visuospatial and attentional paradigms. For example, volleyball players have been shown to respond faster and more accurately than rowers and sedentary controls in visuospatial attention tasks, with performance advantages increasing as a function of competitive level (Giglia et al. 2011; see also Vestberg et al. 2012, Heppe et al. 2016; Heppe and Zentgraf 2019). If these effects reflect training rather than self‐selection, they suggest that open‐skill practice may shape the distribution and flexibility of visuospatial attention. Notably, these advantages appear to extend across the lifespan. Li et al. (2019) demonstrated that older adults engaging in open‐skill exercise exhibited superior inhibition and error‐processing compared to those participating in closed‐skill activities, a finding corroborated by randomized controlled intervention studies showing differential executive‐function improvements following open‐skill versus closed‐skill training in the elderly (Tsai et al. 2017). Together, these results strengthen the argument that open‐skill training can induce training‐related (neurocognitive) adaptations rather than merely reflecting pre‐existing cognitive differences.

At the same time, despite evidence for training‐related benefits across the lifespan, there is increasing evidence that cognitive advantages are neither uniform nor domain‐general. Koch and Krenn (2021) showed that open‐skill athletes outperformed closed‐skill athletes on selected executive‐function measures, but also demonstrated that prior involvement in open‐skill sports predicted executive functioning even among current closed‐skill athletes, suggesting cumulative or transfer effects across sporting careers. Moreover, sport‐specific studies indicate that cognitive advantages often emerge in tasks closely aligned with the perceptual and attentional demands of the sport. For instance, table tennis athletes exhibit selectively enhanced executive attentional control in attention‐focused paradigms rather than across all executive domains (B. Wang et al. 2016), supporting the notion of task‐specific and discipline‐specific cognitive adaptations.

These findings align with earlier work showing that expert athletes in open‐skill sports such as boxing, football, hockey, pentathlon or volleyball display greater attentional flexibility in the spatial orienting of visual attention compared to novices, particularly in their ability to allocate attention toward low‐probability but task‐relevant events (e.g., Enns and Richards 1997; Heppe et al. 2016; Lum et al. 2002; Nougier et al. 1989). In contrast, such strategic modulation of attention appears less pronounced in closed‐skill athletes (Nougier et al. 1996). However, evidence also indicates that athletes from cognitively demanding closed‐skill sports can show advantages in specific executive domains, underscoring that closed‐skill classifications do not necessarily imply low cognitive load. For example, Jacobson and Matthaeus (2014) found that athletes in closed‐skill sports like bowling and running performed better than open‐skill athletes on tasks requiring interference control, although open‐skill athletes had an advantage in problem‐solving tasks. This suggests that certain cognitive functions may be enhanced across both types. Accordingly, several authors have cautioned against overly broad interpretations of the open–closed dichotomy. Heilmann et al. (2022) argued that dichotomous classifications might be too broad to capture the cognitive variability within each category. Their meta‐analysis, which incorporated a more fine‐grained subdivision of skill types along a continuum, found that effect sizes between open‐skill and closed‐skill athletes were small and statistically non‐significant when considering intermediate sport types. They argued that comparing sports with extreme differences—such as gymnastics (a closed skill with high cognitive demand) and team ball sports (a highly dynamic open skill)—may inflate the perceived cognitive advantage of open‐skill athletes. Beyond that, Chueh et al. (2017) who used behavioral and electrophysiological measures, reported enhanced visuospatial performance and neural resource allocation in athletes compared to non‐athletes, but found no systematic differences between open‐skill and closed‐skill sports, suggesting that some cognitive benefits may reflect sport participation per se rather sport‐specific effects. One might also argue that the estimated cognitive differences between sports trace back to relative differences in regular predominant “usage” of cognitive functions rather than differences in manifestation of cognitive functions per se.

Overall, while the body of literature generally supports a cognitive advantage for open‐skill athletes, particularly in dynamic attentional and executive processes, the magnitude and consistency of these effects are influenced by the specificity of the tasks, the nature of the sport, and the way skill categories are defined. Despite the apparent convergence of findings, an important limitation of the existing literature concerns sample size and statistical power. The majority of experimental and cross‐sectional studies comparing open‐skill and closed‐skill athletes rely on relatively small samples, often comprising fewer than 30 participants per group (e.g., Giglia et al. 2011; Nougier et al. 1996; C. H. Wang et al. 2013). Such sample sizes substantially limit the precision of effect‐size estimates and increase susceptibility to both false‐positive and false‐negative findings, particularly when multiple executive‐function outcomes are assessed within the same study. Even meta‐analyses and systematic reviews in this area are largely based on aggregations of underpowered primary studies, which may inflate observed effects and contribute to heterogeneity across age groups, tasks, and sport classifications (Gu et al. 2019; Ren et al. 2025; Zhu et al. 2020). Consequently, while the overall pattern suggests cognitive advantages for athletes engaged in open‐skill sports, the robustness and generalizability of these effects should be interpreted with caution. Creating dichotomous comparisons can lead to the premature conclusion that the results of a pairwise comparison of two sports also applies to two completely different sports also belonging to the open‐skill versus closed‐skill category. It has been suggested that using more differentiated taxonomies may help clarify whether the observed differences are a function of sport type per se or the cognitive demands embedded within particular performance contexts.

##### 1.1.1.2 Strategic, Interceptive, and Static

The tripartite classification of sports into strategic, interceptive, and static has documented differences, particularly in processing speed, attentional control, and executive function. In a meta‐analysis by Voss et al. (2010), athletes in interceptive sports demonstrated the greatest advantage in processing speed tasks compared to those in static and strategic sports. Although the effect sizes for strategic sports were smaller, athletes in these sports often performed better than those in static sports on tasks requiring varied attention and cognitive flexibility, likely reflecting the need for real‐time adaptation to unpredictable team interactions and environmental variables. Krenn et al. (2018) further supported these distinctions in a study employing cognitive inhibition, working memory, and cognitive shifting tasks. Strategic sport athletes showed the fastest reaction times and lowest error rates on congruent and incongruent trials in flanker tasks, suggesting enhanced attentional control and interference suppression. Interceptive athletes also outperformed static athletes on inhibitory tasks but did not match the overall cognitive flexibility observed in strategic athletes. Interestingly, no significant differences were found across sport types in the 2‐back working memory task, indicating that working memory capacity may not be as sport‐type dependent as inhibition or flexibility. Further supporting the strategic–static distinction, Rahimi et al. (2022) found that strategic athletes (i.e., soccer) performed faster and more accurately than static athletes (i.e., track and field) on non‐sport‐related cognitive tasks of the Attention Network Test, particularly under conditions requiring orienting and inhibition of incongruent stimuli. Extending these findings, Yu et al. (2019) showed that strategic sport athletes exhibited higher accuracy but longer reaction times than interceptive athletes in inhibition tasks, accompanied by greater activation of fronto‐parietal control regions, suggesting a stronger reliance on top‐down attentional control in strategic sports. In this regard, Wylie et al. (2018) also found stronger activation of fronto‐parietal regions in strategic athletes and concluded that sport‐type differences may be rather expressed at the level of neural processing strategies and not always manifest in behavioral performance differences (see also Williams and Zentgraf 2025, for a special issue on neuroscience, cognitive function and expertise in sport). Interceptive athletes in a study by N. Yongtawee and Woo (2017) exhibited the shortest reaction times in choice reaction time tasks and superior visuospatial performance, while strategic athletes showed enhanced design fluency and cognitive flexibility on executive function tests. Static athletes generally performed more slowly on reaction time tasks, and in some cases, longer training experience in static sports was associated with delayed processing speed, a finding that contrasts with the usual assumption of performance benefits from expertise. Their follow‐up study (N. Yongtawee and Woo 2021) confirmed previous results. These findings reinforce the idea that cognitive strengths may vary systematically by sport type, with different sports fostering distinct cognitive profiles.

Building on this broader pattern, more targeted comparisons between single disciplines within these categories can further clarify how specific demands shape cognitive skills. Meng et al. (2019) provided direct evidence for the tripartite taxonomy through a comparative study of badminton (interceptive) and volleyball (strategic) players. While badminton players showed faster sensorimotor responses and superior performance in task‐switching speed, volleyball players demonstrated better performance in task‐switching accuracy and alerting components of attention. This suggests that interceptive sports may be particularly associated with rapid visuomotor processing, while strategic sports foster broader executive function capabilities such as cognitive flexibility and proactive attentional control.

Although these studies largely support the cognitive differentiation between strategic, interceptive, and static sports, results are not entirely consistent. For instance, while many studies report strategic and interceptive athletes to outperform static athletes in executive function measures, the specific domain of advantage (e.g., inhibition vs. flexibility vs. processing speed) can vary across studies depending on task type, athlete sample, and sport‐specific training demands. Furthermore, Vona et al. (2024) reported only limited differences in cognitive function across sport categories, noting that in their large‐sample analysis, only one out of twelve cognitive variables—cognitive flexibility—differed significantly, with speed/strength athletes outperforming team sport athletes, a result that does not map neatly onto the strategic‐interceptive‐static framework.

In sum, the strategic‐interceptive‐static taxonomy offers a more nuanced way to explore sport‐related cognitive differences. While converging evidence supports the relevance of this framework, particularly in distinguishing processing speed and executive control capabilities, variability in results suggests that further refinement or sport‐specific adaptation of the taxonomy may be necessary to fully capture the cognitive demands of athletic performance. Awareness of sport‐specific cognitive demands helps ensure that training targets functions pertinent to the discipline, avoiding emphasis on skills more relevant to unrelated sports. A further differentiation might thus be required, as proposed by multidimensional classifications.

##### 1.1.1.3 Multidimensional

To move beyond too narrowly defined classifications, McKay et al. (2021) proposed a multidimensional taxonomy that groups sports into seven broad categories. Although it has not yet been widely applied in cognitive research, its conceptual breadth aligns with the growing recognition that existing dichotomies may oversimplify sport‐specific cognitive demands. Vona et al. (2024), while not explicitly using McKay's categories, reported that team sport athletes showed lower scores on measures of cognitive flexibility compared to athletes in speed/strength sports, although no other cognitive functions differed significantly across sport types. These findings suggest that the cognitive demands of sports with explosive, time‐critical execution may differ from those that require constant environmental monitoring and adaptation. Logan et al. (2022) also indirectly engaged with McKay's categories through their energy systems framework and showed in their meta‐analysis that cognitive performance differed significantly across athlete types. Specifically, aerobic‐trained and HIIT team athletes exhibited stronger performance in attention and executive function tasks compared to controls. Notably, aerobically trained athletes demonstrated the largest overall effect size (*g* = 0.93) for cognitive function, particularly on measures of attentional allocation and cognitive flexibility. HIIT team athletes followed closely (*g* = 0.65), suggesting that intermittent team sports may foster rapid cognitive shifting and stimulus monitoring. Resistance‐trained athletes showed smaller and less consistent cognitive benefits, aligning with the lower cognitive variability typically observed in short‐duration, self‐paced tasks. Their findings suggested that differences in cognitive function—particularly in attention and inhibitory control—are moderated by the type of physiological training athletes engage in, a perspective that reinforces McKay's multidimensional approach.

Despite its promise, the McKay taxonomy has not yet been rigorously tested as a predictive model for cognitive function differences across sport types. One challenge is the within‐category heterogeneity; for instance, “team sports” encompass both basketball and field hockey, which may differ substantially in pacing, player roles, and tactical systems. Moreover, sports such as tennis or judo, and especially multi‐discipline sports like modern pentathlon or decathlon, could reasonably be placed in multiple categories depending on the emphasis of the study (e.g., tactical complexity vs. physical output). McKay et al. ’s (2021) classification system therefore provides a promising structure for organizing comparative research on sport‐specific cognitive function. Its strength lies in the integration of physiological, technical, and contextual dimensions. However, its application to cognitive domains remains in early stages, and future research is needed to examine whether these categories can meaningfully differentiate cognitive profiles, or whether further refinement is required to account for intra‐category variability and cross‐cutting demands.

Summing up, the relationship between sport participation and cognitive function is well supported, yet the consistency and clarity of this link depend heavily on how sports are classified. Binary models such as the open versus closed‐skill distinction point to cognitive advantages for open‐skill athletes, especially in executive domains like flexibility and inhibition. However, the simplicity of this framework may mask important within‐group differences. More refined approaches, such as the tripartite classification of strategic, interceptive, and static sports, offer a clearer view of how specific cognitive skills align with distinct performance demands. Even so, findings vary depending on the cognitive domain measured and the characteristics of the sport or athlete sample. Emerging multidimensional frameworks attempt to capture the complexity of sport‐specific demands by integrating physiological, technical, and contextual factors. These models hold promise but remain underutilized and are challenged by category overlap and heterogeneity, which is why we promote an individualized lens on the relationship between sport and cognitive functioning.

### 1.2 The Individual Lens

Rather than rejecting sport taxonomies outright, an individual lens emphasizes their limitations when used as explanatory models for cognitive performance. One such limitation of taxonomies is their reliance on binary or coarse distinctions that may not capture the rich variability between sports and between sports classified within the same category of a taxonomy. For example, grouping volleyball and basketball together as “externally paced” or “strategic” overlooks key differences in tempo, player roles, and perceptual‐motor demands. Similarly, labeling sports like gymnastics and long‐distance running as “static” or “closed skill” does not account for their distinct demands on spatial awareness, timing, or psychological regulation. Even attempts to integrate physiological profiles (e.g., aerobic vs. anaerobic training) or interaction dynamics (e.g., combat vs. object sports) often fail to reflect the full cognitive complexity experienced by athletes in competition. Moreover, cognitive performance outcomes associated with these groupings are inconsistent across studies. While some research finds that athletes in open‐skill or interceptive sports show superior executive functioning (e.g., inhibitory control or processing speed), other studies report negligible or contradictory results. These inconsistencies suggest that broad taxonomies may not be sufficiently sensitive to differentiate between specific sport demands or to explain individual differences in cognitive function among athletes.

Importantly, the absence of consistent behavioral differences across sport types cannot be interpreted as unequivocal evidence that cognitive demands do not differ meaningfully between sports or that taxonomic classifications lack explanatory value. An alternative explanation, increasingly emphasized in recent neurocognitive research, is that commonly used behavioral measures—such as reaction time and accuracy—may lack sensitivity in elite athlete samples due to ceiling effects and performance optimization. In highly trained populations, athletes may converge on similarly high levels of behavioral performance despite relying on distinct underlying neurocognitive processes. Supporting this interpretation, recent studies comparing elite athletes from different sport disciplines have reported comparable behavioral performance in tasks assessing working memory and inhibitory control, while simultaneously revealing differences in neural activation patterns and processing strategies (e.g., Yao et al. 2024). Similarly, electrophysiological evidence indicates that athletes can differ in neural markers of cognitive control despite showing indistinguishable behavioral outcomes (e.g., Yao, Fu, et al. 2024). These findings suggest that sport‐specific adaptations may manifest at the level of neural efficiency, recruitment, or strategy rather than overt behavioral performance. Consequently, null findings in behavioral paradigms should be interpreted with caution, as they may obscure meaningful neurocognitive differences that are more closely aligned with the demands captured by sport taxonomies.

In this study, we used data from a unique sample of national squad athletes across eight sport disciplines (artistic gymnastics, basketball, ice hockey, modern pentathlon, rhythmic gymnastics, table tennis, trampoline, volleyball) to examine whether established sport taxonomies provide a meaningful framework for differentiating cognitive performance across sports. Importantly, our analyses are based on standardized, domain‐general cognitive tasks, which represent the predominant methodological approach in the existing literature. While such tasks enable comparability across studies and cognitive domains, they may be less sensitive to subtle, sport‐specific adaptations in highly trained populations. Therefore, rather than interpreting the absence of behavioral differences as evidence for the absence of underlying cognitive distinctions, the present study evaluates whether commonly used taxonomic classifications account for measurable variance in behavioral cognitive performance under typical assessment conditions.

## 2 Method

### 2.1 Data Acquisition

This cross‐sectional data set was acquired between February 2022 and April 2025 and was part of a larger multi‐disciplinary research project (Zentgraf et al. 2024). Since cognitive diagnostics of this large‐scale project were partly reformed after 2 years and some tasks were introduced later, sub sample sizes of the different tasks vary (see Table 2).

**TABLE 2:** Sub sample sizes for each cognitive measures divided by gender and by sport discipline.

	ZVT	d2‐R	Backw. Corsi	N‐L‐T	Flanker
Total *N*	567	379	473	326	328
Male/Female	320/247	186/193	271/202	200/124	202/126
Basketball	75	40	62	55	56
Volleyball	177	138	174	105	105
Ice hockey	126	67	73	64	62
Artistic gymnastics	39	22	33	28	28
Rhythmic gymnastics	32	29	29	17	19
Trampoline	46	33	40	33	33
Table Tennis	40	18	36	22	24
Modern pentathlon	32	32	26	0	0

We refer to our participants as elite athletes, because all athletes were tested at junior or senior national team training camps for which they had been explicitly nominated by their national coaches, yielding a heterogeneous national‐squad population (for further expertise differentiation, please see Zentgraf et al. 2024). At the beginning of each diagnostic day, participants completed a cognitive group task (i.e., trail‐making task, see measures below) and were then randomly allocated to different test stations measuring motor functioning (i.e., strength, power, postural stability, mobility, speed, etc.), blood health (i.e., micronutrients, inflammation, genetic composition, hormones etc.), gut health, psychosocial and environmental factors, as well as cognitive functions. Data from the latter is at the core of this article.

### 2.2 Measures

Cognitive functions were differentiated into basic (processing speed, attention) and higher CFs (working memory, inhibition, cognitive flexibility) in line with recent empirical work (Kalén et al. 2021; Zentgraf et al. 2024). Variables of basic CF tasks were standardized by age‐matched norms from the German population. Our classification followed a pragmatic, task‐dominant perspective: both basic CF tasks partly involve executive components, but were classified as “basic” due to their dominant reliance on single‐function efficiency rather than coordination across multiple control processes (e.g., ZVT was treated as a measure of basic processing speed because it primarily captures speeded serial information processing despite requiring executive pacing; Rammsayer and Stahl 2007).

#### 2.2.1 Basic Cognitive Functions

##### 2.2.1.1 Processing Speed

In a numbered trail‐making task (Oswald 2016), participants were instructed to prioritize speed over neatness, and completed four pages connecting numbers from 0‐90 within 30 s/page. The task was completed as a group test with all athletes. The average number of connections across all pages represented information processing speed (i.e., the more the higher). Raw values were transformed into standardized values according to the test manual, representing the main dependent variable.

##### 2.2.1.2 Attention

In the electronic d2‐R test (Brickenkamp et al. 2010), participants crossed out d's with two dashes while ignoring irrelevant stimuli (p's & d's with one dash) over 14 test screen pages (60 objects each) within 20 s/page. The concentration score (i.e., correctly marked targets—wrongly marked objects = speed‐accuracy tradeoff) represented selective attention. Its raw value was transformed into an age‐matched standard value according to the test manual's norms.

#### 2.2.2 Higher Cognitive Functions

##### 2.2.2.1 Working Memory Capacity (WMC)

The Backward Corsi Block Tapping test assessed visual‐spatial WM. Participants viewed squares lighting up in sequence and had to recall and click them on a touch screen in reverse order. Sequence length increased after every two successful rounds (min 2, max. 8 squares). The task ended after two failed attempts, and the span score (min‐max: 0‐8; longest correct sequence) indicated WM capacity.

##### 2.2.2.2 Inhibition

A letter version of the Eriksen Flanker task as available in the PsyToolkit library (https://www.psytoolkit.org/experiment‐library/flanker.html) assessed response inhibition and flexibility. Participants responded to the identity of a central target letter (X, C, V, B) that was flanked on both sides by either congruent or incongruent letters. For example, in a congruent trial (e.g., XXXXX), the flanking letters matched the target, while in incongruent trials (e.g., VVCVV), the flanking letters differed. Participants were instructed to respond as quickly and accurately as possible using predefined keys (left key: X, C; right key: V, B), while ignoring the flanking letters. Response inhibition is considered high, the lower the difference in reaction time in incongruent compared to congruent trials (i.e., the Flanker effect).

##### 2.2.2.3 Cognitive Flexibility

Task Switching was assessed by a number‐letter‐task, also available through the PsyToolkit platform (https://www.psytoolkit.org/experiment‐library/taskswitching.html). In this task, a number‐letter pair (e.g., G7) appears in one of four quadrants on the screen. Depending on the quadrant, participants are required to perform one of two tasks: (a) categorize the letter as a vowel or consonant, or (b) categorize the number as odd or even. The position of the stimulus determines which task to perform, requiring participants to alternate between tasks on successive trials. The task includes both repeat trials (same task as previous trial) and switch trials (different task from previous trial). Switch costs are calculated as the performance difference between switch and repeat trials, reflecting the cognitive demands of shifting mental sets.

### 2.3 Participants

In total, *N* = 595 athletes were part of the multidisciplinary diagnostics (*M*_age_ = 18.01 ± 5.36 years), of which 255 were female and 340 were male. The different sport disciplines were represented as follows: 6.72% artistic gymnastics, 7.73% trampoline, 5.38% rhythmic gymnastics, 14.29% basketball, 21.85% ice hockey, 7.06% table tennis, 31.60% volleyball, 5.38% modern pentathlon.

As outlined earlier, due to changes in diagnostics over the course of the project, sub sample sizes varied. After removing outlier from the data (i.e., RT in switch trials (number‐letter task) and incongruent trials (flanker) > 2000 ms; Corsi span = 0), the division of n's was as outlined in Table 2.

### 2.4 Data Analysis

To examine whether taxonomic classifications meaningfully account for variance in cognitive performance across sports, a model comparison approach was employed. For each cognitive function (processing speed, attention, working memory, inhibition, and cognitive flexibility), a series of generalized linear models were estimated. Specifically, five competing models were specified: (a) a null model including only the intercept, (b) a model including sport discipline as a categorical predictor (eight levels), and (c–f) four separate models including one taxonomy each as a categorical predictor (open vs. closed; strategic vs. static vs. interceptive; participant classification framework; team vs. precision‐skill vs. speed‐strength). Each taxonomy represents a reclassification of the same underlying sport variable into broader categories, see Table 3. All models were estimated using identical datasets for each outcome variable to ensure comparability. Model fit was evaluated using Akaike's Information Criterion (AIC) and Bayesian Information Criterion (BIC), with lower values indicating better model fit. Differences in AIC (ΔAIC) were used to assess the relative support for competing models, with values greater than 10 indicating substantial differences in model fit.

**TABLE 3:** Sport taxonomies and the factorial classification.

Single disciplines	Open/closed	Strategic/static/interceptive	Participant classification framework	Team/precision‐skill/speed‐strength
Basketball (1)	Open (1)	Strategic (1)	Team (1)	Team (1)
Volleyball (2)	Open (1)	Strategic (1)	Team (1)	Team (1)
Ice hockey (3)	Open (1)	Strategic (1)	Team (1)	Team (1)
Artistic gymnastics (4)	Closed (2)	Static (2)	Precision/skill‐d. (2)	Precision/skill‐d. (2)
Rhythmic gymnastics (5)	Closed (2)	Static (2)	Precision/skill‐d. (2)	Precision/skill‐d. (2)
Trampoline (6)	Closed (2)	Static (2)	Precision/skill‐d. (2)	Precision/skill‐d. (2)
Table Tennis (7)	Open (1)	Interceptive (3)	Racquet (3)	Precision/skill‐d. (2)
Modern pentathlon (8)	Closed (2)	Interceptive (3)	Middle dist./power (4)	Speed‐strength (3)

*Note:* The number in brackets indicates the fixed factor.

Abbreviation: skill‐d, skill‐dependent.

Multilevel (mixed‐effects) models with athletes nested within sports were initially explored to account for the hierarchical structure of the data. For basic cognitive function, that is, processing speed and attention, these models could be estimated without singularity problems when specified with a random intercept for sport discipline. In contrast, for working memory, inhibition and cognitive flexibility, singular fits occurred already in the null models, indicating that the sport‐level random effect could not be estimated reliably for these outcomes. Because the random‐effects structure was therefore not robust across all cognitive functions, the primary analyses were conducted using a fixed‐effects model comparison framework across all dependent variables. Including multiple taxonomies simultaneously in the same model would have introduced perfect or near‐perfect collinearity and no independent variance, as they are derived from the same underlying classification, and would therefore not have provided independent explanatory information. The fixed‐effects model comparison allows for a direct evaluation of the explanatory value of alternative classification systems without imposing an unsupported hierarchical structure. Once a single‐discipline model was more plausible and might therefore yield more nuanced insights where cognitive differences occur as, we further showcase exemplary one‐way ANOVAs while controlling for type 1 error.

Beyond that, norm tables of the original manual of the ZVT were consulted to transform standardized processing speed values into IQ scores. This means, IQ scores were derived from the same ZVT measure and do not represent an independent intelligence assessment. We used this conversion as an interpretability aid to contextualize the magnitude of standardized ZVT differences relative to commonly understood norm metrics. The IQ‐referenced scores were therefore compared against the general population's mean (i.e., 100) and Pearson correlations were calculated to examine whether a higher IQ‐referenced score was related to higher basic and higher cognitive functions.

In addition, Euclidean distances for all available data points were calculated, once separately for each cognitive function, to examine whether data points are generally spread across all individuals. Second with “discipline” as a grouping factor, to examine how much data points were away from each other within isolated sports disciplines. The Euclidean distance analysis was not intended to identify latent cognitive structures, but to quantify the magnitude of interindividual variability relative to group‐level effects. The R code for Euclidean distances is available in the supplementary material.

## 3 Results

The aim of this study was to examine whether sport taxonomic classifications meaningfully differentiate cognitive functions across different sport disciplines. Model comparisons were conducted for each cognitive outcome to evaluate the explanatory value of sport disciplines and competing taxonomic classifications. For each outcome, a null model, a sport‐discipline model, and four taxonomy‐based models were estimated and compared using Akaike's Information Criterion (AIC) and Bayesian Information Criterion (BIC), with lower values indicating better model fit.

Exploratory mixed‐effects models indicated that a random intercept for sport could not be estimated reliably for several outcomes, as singular fits occurred already in the null models for working memory, inhibition, and cognitive flexibility. Therefore, all results reported below are based on fixed‐effects model comparisons to ensure a consistent analytical framework across outcomes (see Table 4). Across cognitive functions, single sport disciplines provided the best model fit for processing speed and working memory, whereas taxonomy‐based classifications did not improve model fit relative to the null model. For attention, all models performed similarly, and for inhibition and cognitive flexibility, no meaningful structure was observed. Overall, these findings provide little support for the explanatory value of existing sport taxonomies in differentiating cognitive performance.

**TABLE 4:** Results of model comparison approach contrasting AIC and BIC.

Model	Processing speed			Attention			Working memory			Inhibition			Cognitive flexibility		
	N	AIC	BIC	N	AIC	BIC	N	AIC	BIC	N	AIC	BIC	N	AIC	BIC
Null model	567	4203.60	4216.60	379	2754.10	2765.90	492	1488.56	1501.04	328	3916.65	3928.03	324	4445.87	4457.21
Single disciplines		*4194.80	4238.20		2751.30	2790.70		1493.16	1534.75		3923.27	3957.40		4448.68	4482.71
Open versus closed		4204.50	4221.90		2751.10	2769.86		1490.52	1507.16		3918.36	3933.53		4447.84	4462.96
Strategic versus static versus interceptive		4207.00	4228.70		2757.38	2777.07		1488.41	1509.20		3919.92	3938.89		4449.80	4468.71
Participant classification framework		4222.50	4291.90		2769.32	2832.32		1509.60	1576.15		3921.15	3973.88		4449.80	4468.71
Team versus precision‐skill versus speed‐strength		4207.16	4228.86		2752.67	2772.36		1490.71	1511.50		3917.98	3933.16		4447.81	4462.93

*Note:* asterisk indicates best plausible model.

### 3.1 Processing Speed

The model including single sport disciplines provided the best fit to the data (AIC = 4194.8), outperforming both the null model (AIC = 4203.6; ΔAIC = 8.8) and all taxonomy‐based models (ΔAIC ≥ 9.7). None of the taxonomic classifications improved model fit relative to the null model.

### 3.2 Attention

For attention, model fit was comparable across the single discipline model (AIC = 2751.3), the open versus closed classification (AIC = 2751.1), and the null model (AIC = 2754.1), with all differences in AIC below 3. This indicates only weak and non‐specific structure across classification systems.

### 3.3 Working Memory

For working memory, no model provided a meaningful improvement over the null model (AIC = 1488.6). Although the strategic versus static versus interceptive classification yielded the lowest AIC (1488.4), the difference relative to the null model was negligible (ΔAIC < 1), indicating no substantive explanatory value.

### 3.4 Inhibition

For inhibition, the null model provided the best fit (AIC = 3916.7), with all alternative models yielding comparable or worse fit (ΔAIC < 2). This indicates that neither sport disciplines nor taxonomic classifications accounted for meaningful variance.

### 3.5 Cognitive Flexibility

Similarly, for cognitive flexibility, the null model provided the best fit (AIC = 4614.1), and no model improved upon it (ΔAIC < 2).

### 3.6 Exemplary Look at Differences Between Sport Disciplines for Processing Speed

As the model comparison approach demonstrates, only processing speed showed clear differentiation at the level of sport disciplines, whereas taxonomy‐based classifications did not provide meaningful explanatory value. These findings provide little support for the assumption that existing sport taxonomies capture systematic differences in cognitive performance across sports. For processing speed, however, differences were found and as such we will use this cognitive function as an example to illustrate how these differences manifest in our sample and how specifically taxonomies would have obscured them.

**FIGURE 1:**
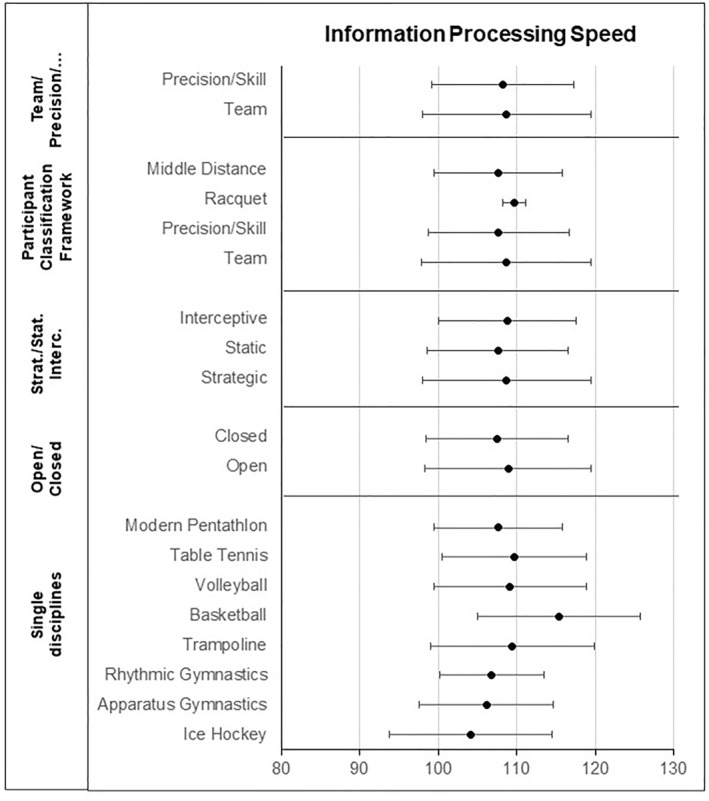
Comparing standardized values in paradigms measuring processing speed across single sport disciplines and different taxonomies.

Using a one‐way ANOVA (8 levels) on the effect of single discipline on processing speed, we found a significant effect of discipline on processing speed (ZVT), *F*(7, 559) = 9.77, *p* < 0.001, *η* ^2^ = 0.109, indicating a moderate effect size. Polynomial contrast analysis revealed significant quadratic (*p* = 0.011, *d* = 0.38), cubic (*p* < 0.001, *d* = −0.55), quartic (*p* = 0.002, *d* = 0.42), and sextic (*p* = 0.023, *d* = −0.32) trends across the sport disciplines. The linear trend did not reach significance (*p* = 0.083, *d* = −0.25). Post‐hoc comparisons using Bonferroni correction (corrected for comparing a family of 28) revealed that participants from Basketball (*M* = 115.36, SD = 10.37) had significantly higher standard values in processing speed than several other disciplines: Artistic (*M* = 106.15, SD = 8.58) and Rhythmic Gymnastics (*M* = 106.84, SD = 6.60), *p* < 0.001 (*d* = 0.95 and *d* = 0.88, respectively), Trampoline (*M* = 109.46, SD = 10.49), *p* = 0.033, *d* = 0.61, Ice Hockey (*M* = 104.17, SD = 10.41), *p* < 0.001, *d* = 1.16, Modern Pentathlon (*M* = 107.69, SD = 8.17), *p* = 0.005, *d* = 0.79, and Volleyball (*M* = 109.18, SD = 9.66), *p* < 0.001, *d* = 0.64. Additionally, Ice Hockey athletes scored significantly lower compared to Table Tennis (*M* = 109.70, SD = 9.13), *p* = 0.048, *d* = −0.57 Trampoline, *p* = 0.044, *d* = −0.55 and Volleyball, *p* < 0.001, *d* = −0.52. None of the taxonomic classification would have suggested differences, all *F* < 3, *p*'s > 0.14. See Figure 1 for all results visualized.

#### 3.6.1 IQ‐Referenced Scores

**FIGURE 2:**
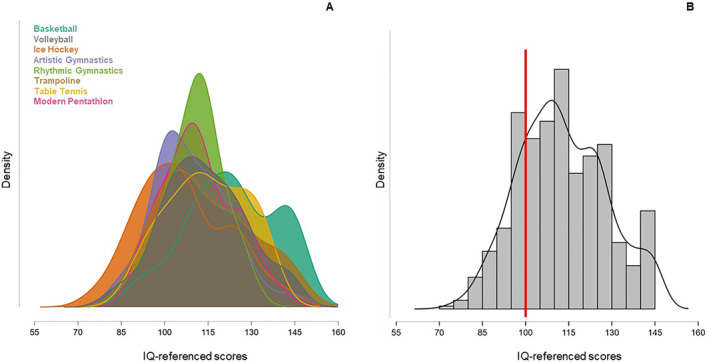
Density curves of IQ‐referenced scores of the different sport disciplines (A) and the full sample (B) of 567 elite athletes. The red line in (B) marks the standardized population mean of 100.

The significant differences in basic cognitive functions between the single disciplines prompted us to contextualize their magnitude by commonly understood norm metrics. The norm values obtained in the processing speed measure (i.e., ZVT) allow a cross‐reference to IQ‐values without having actually conducted full‐fledged intelligence test batteries. Using conversion tables of the official manuscript (Oswald 2016), we transformed standardized values into IQ‐referenced values and found that, overall, elite athletes score significantly above the general population mean of 100 with *M* = 112.78 (SD = 15.28), *t*(566) = 19.91, *p* < 0.001, *d* = 0.836 (see Figure 2, B). There was also a significant effect of sport type on IQ, *F*(7, 599) = 9.77, *p* < 0.001, *η* ^2^ = 0.109 (see Figure 2, A, for differences across sport types and for post‐hoc comparisons see Supporting Information S1: appendix Table X1). IQ‐referenced scores (i.e., processing speed norms) significantly correlated moderately with attention, *r*(369) = 0.43, *p* < 0.001, 95% CI [0.34, 0.51], *z* = 0.457, and weakly with working memory capacity, *r*(465) = 0.20, *p* < 0.001, 95% CI [0.11, 0.27], *z* = 0.203, suggesting shared variance between norm‐referenced processing speed and performance on these tasks. Correlations to inhibition, *r*(310) = 0.04, *p* = 0.469, *z* = 0.041, and cognitive flexibility were negligible, *r*(315) = 0.04, *p* = 0.541, *z* = 0.035.

#### 3.6.2 Euclidean Distances

Unlike variance estimates within predefined groups, Euclidean distances provide a task‐comparable metric of how dissimilar any two athletes are, independent of taxonomic assignment, thereby directly addressing whether within‐group variability rivals or exceeds between‐group differences. After z‐standardizing all variables, pairwise Euclidean distances were calculated for each cognitive variable. Distances are expressed in standard deviation (SD) units, making them directly comparable across measures. Mean distances ranged from 1.08 SDs (working memory) to 1.14 SDs (processing speed) with median values between 0.88 and 0.98 SDs, indicating that, on average, any two athletes differed by about one SD in these tasks. Minimum distances were 0.0 SDs for all variables, reflecting instances of identical or near‐identical performance between some athletes. In contrast, maximum distances reached up to 9.13 SDs (cognitive flexibility) and 6.90 SDs (cognitive inhibition) in the higher cognitive function tasks, highlighting athlete pairs with markedly divergent performance profiles. Among the assessed tasks, working memory showed the smallest average distance (1.08 SDs), suggesting relatively tighter clustering of scores, whereas the basic cognitive function measures (i.e., processing speed and attention) exhibited slightly higher average distances (1.14 and 1.13 SDs, respectively), indicating marginally greater dispersion. The combination of low minimums and high maximums, together with means consistently above 1.0 SD, points to substantial interindividual variability across all assessed cognitive domains. These findings imply that even within an elite sample, athletes exhibit both clusters of high similarity and substantial performance gaps, which may have practical implications for individualized cognitive training or talent identification.

Across disciplines, mean within‐group distances for the filtered and z‐standardized variables typically ranged around 0.8–1.4 SDs, indicating moderate performance variability within teams. The tightest clustering was observed in Rhythmic Gymnastics and Table Tennis for working memory (means ≈ 0.81 SDs), suggesting relatively homogeneous cognitive performance in these groups. In contrast, Artistic Gymnastic showed the largest within‐group spread on this variable (mean = 1.43 SDs), pointing to more heterogeneous athlete profiles. Maximum distances reached over 5 SDs in some disciplines (e.g., Basketball, Artistic Gymnastics), which reveals that even within a single sport, there are athlete pairs with highly divergent cognitive performance (see Supporting Information S1: appendix Table X2). These patterns underline that intra‐discipline variability can be substantial, with potential implications for tailoring sportpsychological diagnostics and interventions to individual athletes.

## 4 Discussion

The aim of this study was to assess whether different sport taxonomies meaningfully differentiate cognitive functions among elite athletes. Using a comparably large data set of junior and senior national squad athletes, from a large multi‐center study with access to different sports completing the very same cognitive tasks, enabled a systematic comparison under realistic elite‐sport sampling conditions.

Under the present sampling conditions (e.g., many athletes yet also unequal sub‐sample sizes, sport‐linked age and gender compositions) measuring performance on domain‐general paradigms, only processing speed showed a meaningful structure, with the single‐discipline model providing the best fit. In contrast, taxonomy‐based models did not improve model fit relative to the null model. For attention, working memory, inhibition, and cognitive flexibility, all models showed comparable fit or failed to outperform the null model, indicating weak or absent structure across both sport and taxonomy‐based classifications.

These findings challenge the assumption that established sport taxonomies provide a robust and generalizable framework for differentiating cognitive performance (e.g., Gu et al. 2019; Voss et al. 2010). Classifications such as open versus closed skills, strategic–static–interceptive distinctions, or participant classification frameworks did not account for meaningful variance in cognitive outcomes under the present conditions. Importantly, even when small numerical differences between models were observed (e.g., for attention), these differences were negligible in magnitude (ΔAIC < 2) and therefore do not support substantive interpretation. This contrasts with previous meta‐analytic findings suggesting advantages for open‐skill athletes in attention, inhibition, and cognitive flexibility (Ren et al. 2025; Zhu et al. 2020). The only domain showing consistent differentiation was processing speed, where individual sport disciplines clearly outperformed all taxonomic classifications. This may suggest that certain basic cognitive functions are more closely tied to sport‐specific demands, which is consistent with prior work reporting sport‐related differences in processing speed and attention (e.g., A. Yongtawee et al. 2021; Krenn et al. 2018). It is also possible that in our specific sample, ceiling effects occurred and that our sample was above‐average in cognitive function per se. While elite athletes have been reported to demonstrate superior cognitive functions, small to moderate evidence for higher intelligence remains however mixed (Voss et al. 2010; Mitić et al. 2021). At the same time, the absence of systematic effects for higher‐order cognitive functions aligns with studies reporting limited or inconsistent differences in executive functions across sport types (Vona et al. 2024; Logan et al. 2022).

Importantly, null effects in higher cognitive functions should not be interpreted as evidence that executive control is equivalent across sports, but rather as reflecting limited sensitivity of domain‐general tasks, substantial interindividual variability, and the possibility that sport‐specific adaptations manifest primarily at the level of neural processing rather than overt behavioral performance. This interpretation is consistent with the argument that overly coarse or binary classifications may obscure within‐category variability and inflate perceived differences when only extreme cases are compared (Heilmann et al. 2022). This perspective is further supported by the observed within‐discipline variability. Even after removing outliers and standardizing scores, cognitive performance differences within sports were substantial, indicating that heterogeneity at the individual level may overshadow between‐group differences. Our additional norm‐referenced interpretation of processing speed may speak for differences in cognitive prerequisites (i.e., ZVT baseline); however, they were not (or only to a very small degree) related to executive control and seem to be generally well‐developed, considering that elite athletes, on average, exhibit higher intelligence‐referenced values than the general population. Because these IQ‐equivalent values were a linear transformation of ZVT scores, they were, however primarily reported for interpretability and should not be interpreted as evidence about general intelligence. In addition, our within‐discipline Euclidean distance analyses suggest that cognitive performance variability among athletes in the same sport can be substantial. With these analyses we intended to describe the magnitude of interindividual variability rather than to model cognitive structure. Even after removing outliers and z‐standardizing scores, mean within‐group distances typically ranged from about 0.8 to 1.4 SDs, with maxima exceeding 5 SDs in some cases. This means that while some disciplines, such as sport gymnastics or table tennis, showed tightly clustered performance profiles, others (e.g., apparatus gymnastics, basketball) contained athlete pairs with markedly divergent cognitive abilities. Such within‐sport variability also aligns with the notion that athletes cannot always be reduced to a single, stable sport‐specific profile, because also previous multidimensional experience contributes to heterogeneity among athletes. Athletes cannot always be reduced to a single sport‐specific profile, as their skills may extend across multiple sports with varying levels of proficiency. For instance, an athlete who performs at an elite level in basketball may show moderate competence in soccer and lower performance in table tennis. Exceptional cases such as Eddie Eagan (winning Olympic gold medals in boxing and bobsleigh) or Alexandra Burghardt (Olympic success in athletics and later in bobsleigh) illustrate that high‐level performance can even occur across markedly different sports. While such examples are rare, they highlight that sport‐specific categorization may oversimplify the broader structure of athletic ability. This raises the question of whether sports can be meaningfully classified solely based on cognitive or task‐related demands, or whether more individualized perspectives on athlete capabilities should be considered.

This interpretation is further supported by recent neurocognitive evidence suggesting that the absence of behavioral differences does not imply equivalent underlying processing. Studies on elite athletes have demonstrated that comparable performance in domain‐general tasks (e.g., working memory or inhibition) can be accompanied by distinct neural activation patterns and processing strategies across sport disciplines. For example, Yao et al. (2024) reported no behavioral differences between Olympic‐level athletes from different sports, while identifying sport‐specific functional brain activation profiles. Similarly, electrophysiological findings indicate differences in neural markers of inhibitory control despite identical behavioral outcomes (e.g., Yao, Fu, et al. 2024). These results suggest that sport‐related cognitive adaptations may manifest in neural efficiency, resource allocation, or strategy selection rather than in overt performance metrics. From this perspective, sport taxonomies may still capture meaningful differences in underlying neurocognitive demands, even when such differences are not detectable using standard behavioral paradigms. Accordingly, the present findings should be interpreted as reflecting the limited sensitivity of domain‐general behavioral tasks to these adaptations, rather than as evidence against the existence of sport‐specific cognitive specializations.

Taken together, the findings suggest that cognitive differences in elite sport are neither consistently structured by taxonomic classifications nor uniformly aligned with sport disciplines across all cognitive domains. Instead, the results point toward a domain‐specific and relatively weak structure, where only certain basic cognitive functions (e.g., processing speed) show reliable sport‐related differentiation. From a methodological perspective, the present study highlights the importance of directly comparing competing classification frameworks rather than testing them in isolation. The model comparison approach used here provides a more stringent test of theoretical assumptions by evaluating whether taxonomies meaningfully improve model fit relative to both sport‐level models and null models. In addition, exploratory mixed‐effects analyses indicated that sport‐level variance components could not be estimated reliably for several outcomes, further underscoring the limited robustness of hierarchical structure in the present data.

Several limitations should be considered. It is important to acknowledge that the disciplines examined in this study constituted a convenience sample from a larger research project. As such, they may not capture the full range of variability across sports, particularly given that some are relatively similar in nature (e.g., artistic gymnastics and trampoline). Furthermore, age, which can be sensitive to executive function in terms of developmental differences, varied across disciplines as athletes' peak performance age depends on discipline and competition levels. With certain sports like rhythmic gymnastics versus table tennis differing significantly in peak performance age (e.g., Longo et al. 2016), some age variance was present in our dataset, still age was not included as a control variable in the analyses. Age correction was not feasible because standardized age‐based norms for the specific tasks used in this study are currently unavailable for both normal and athlete populations. Without such norms, statistically controlling for age would require imposing assumptions about the nature of age effects that cannot be empirically justified within the present measurement framework. Moreover, the primary objective of the study was to compare patterns of executive functioning across sport types rather than to estimate age‐adjusted cognitive ability. In addition, not all disciplines contributed data to all cognitive measures (e.g., modern pentathlon), which further limits the comparability of taxonomic contrasts. However, the contrast between results from single disciplines versus taxonomic groups is particularly revealing. Processing speed and attention differed significantly between sports such as basketball and table tennis versus gymnastics or modern pentathlon—findings that resonate with prior work (e.g., A. Yongtawee et al. 2021; Krenn et al. 2018). These differences were not detectable when athletes were aggregated into broader taxonomic categories. For instance, while basketball consistently outperformed gymnastics on processing speed, both were labeled as either “strategic” or “closed‐skill” depending on the taxonomy. Taking away sport‐specific nuances but creating more generic labels concealed meaningful cognitive contrasts.

Even the more nuanced tripartite taxonomy (strategic, static, interceptive), which has previously shown promise in differentiating cognitive traits such as attention and inhibition (Voss et al. 2010; Meng et al. 2019), did not yield significant distinctions in our study. One possible explanation is that many real‐world sports are hybrid and elude neat categorization. For instance, volleyball and basketball are both labeled as strategic team sports, yet they differ considerably in tempo, tactical structure, and cognitive load distribution across positions. The same holds for ice hockey and table tennis—despite being classified under the same open‐skill or strategic umbrellas, their demands on sensorimotor processing, anticipation, and tactical thinking vary profoundly. It should be noted, however, that uneven sub‐sample sizes and overlapping sport characteristics may have reduced statistical power to detect such distinctions.

Taken together, our results suggest that while classification systems offer a convenient heuristic for organizing research and simplifying data analysis, they may be of limited utility when used as explanatory models for individual cognitive profiles in elite sport. The present findings should not be interpreted as definitive evidence against the validity of sport taxonomies per se, but rather as an illustration of their limited robustness and sensitivity under realistic empirical constraints. Yet, to validly reflect the cognitive demands of a specific sport is especially warranted in a high‐performance setting. There, athletes and coaches aim to maximize the individual potential of an athlete to make him/her excel within their sport; a comparison to other sports is irrelevant. As also noted by Zentgraf et al. (2024), the complexity of elite performance is unlikely to be captured by taxonomic groupings alone. Instead, we advocate for a more individualized, discipline‐specific lens that recognizes the embedded cognitive demands unique to each sport and athlete.

This approach aligns more closely with current developments in personalized training and diagnostics in elite sport. For instance, a meta‐analysis by Kalén et al. (2021) pointed out that beyond domain‐general cognitive measures, specifically sport‐specific task stimuli yield larger effects in differentiating higher‐from lower‐skilled athletes. With far‐transfer effects from domain‐general diagnostics to sport‐specific demands being limited, one promising direction in cognitive diagnostics is thus the development of sport‐specific variants of the classic cognitive tasks to increase the representativeness of the methods employed (e.g., exchanging letters and numbers in a task‐switching paradigm in jersey colors and numbers in ice hockey). Also promising, as already under continuous development, is the use of sport‐specific decision tasks and option‐generation paradigms with temporal or spatial occlusion (e.g., Musculus et al. 2019). Practically, cognitive diagnostics in elite sport should not rely solely on generalized assumptions derived from broad sport types. Instead, assessments should be tailored to the specific perceptual, attentional, and decision‐making demands of the athlete's discipline and role within the team (e.g., Kalén et al. 2021; Musculus et al. 2019). This individualized approach not only improves ecological validity but may also help identify cognitive strengths and needs that can inform training design and performance support.

To conclude, this study provides empirical evidence from an exceptionally large sample of elite athletes that sport taxonomies—while conceptually appealing—may not reliably differentiate cognitive function among elite athletes when applied to heterogeneous samples, domain‐general tasks, and uneven sport representations. Accordingly, our findings speak primarily to robustness rather than to the existence (or absence) of sport‐specific cognitive adaptations. Differences in cognitive performance appear more readily at the level of individual disciplines rather than across abstracted categories, and substantial variability exists even within a given sport. Importantly, these conclusions are restricted to behavioral manifestations of cognitive performance and do not preclude the existence of sport‐specific adaptations at the neurocognitive level. These results support a growing body of literature advocating for an individualized, context‐sensitive approach to studying cognition in sport and raise critical questions about the utility of broad taxonomic classifications as explanatory models in both research and applied settings.

## Funding

This research was funded by the Bundesinstitut für Sportwissenschaft (German The Federal Institute of Sport Science) in 2021–2028 (Grant No. 081901/21‐28, German: Individuelle Leistungsentwicklung im Spitzensport durch ganzheitliche und transdisziplinäre Prozessoptimierung; English: Individual performance development in elite sports by holistic and transdisciplinary process optimization).

## Ethics Statement

The study protocol was approved by the Ethics Committee of the Justus Liebig University Giessen (ethical approval number: AZ 55/22; approval date: 10 May 2022).

## Consent

Participants provided written informed consent to participate in accordance with the Declaration of Helsinki before taking part in any diagnostic in the larger‐scale project.

## Conflicts of Interest

The authors declare no conflicts of interest.

## Permission to Reproduce Material From Other Sources

The authors have nothing to report.

## Supporting information


Supporting Information S1

## Data Availability

Data cannot be made fully available publicly because this could lead to elite athletes being identified. However, a subset of the data as z score means with standard deviations that is deidentified can be obtained on demand from the corresponding author.
