# Supplementary material for: A Critical Examination of the Usefulness of Taxonomies for Comparing Cognitive Functions Across Sports
Source: Eur J Sport Sci. 2026 Jun 19;26(7):e70209. doi: 10.1002/ejsc.70209 (PMC13282182; doi:10.1002/ejsc.70209)
Supplement: Supplementary file 1 — Supporting Information S1 [file EJSC-26-e70209-s001.docx]

**Supplementary Material**

**Table X1**

*Post-hoc Comparisons of ANOVA examining differences in IQ across sport disciplines*

| *Post-hoc Comparisons - discipline* | | | | | | | | | | | | | |
| --- | --- | --- | --- | --- | --- | --- | --- | --- | --- | --- | --- | --- | --- |
|  | |  | | Mean Difference | | SE | | t | | Cohen's d | | p_tukey_ | |
| **Basketball** |  | **Artistic Gymnastics** |  | **13.809** |  | **2.865** |  | **4.820** |  | **0.951** |  | **< .001** | ******* |
|  |  | **Ice Hockey** |  | **16.790** |  | **2.117** |  | **7.932** |  | **1.157** |  | **< .001** | ******* |
|  |  | Table Tennis |  | 8.490 |  | 2.842 |  | 2.988 |  | 0.585 |  | 0.058 |  |
|  |  | **Trampoline** |  | **8.855** |  | **2.718** |  | **3.258** |  | **0.610** |  | **0.026** | ***** |
|  |  | **Rhythmic Gymnastics** |  | **12.774** |  | **3.064** |  | **4.169** |  | **0.880** |  | **< .001** | ******* |
|  |  | **Volleyball** |  | **9.269** |  | **2.000** |  | **4.635** |  | **0.639** |  | **< .001** | ******* |
|  |  | **Modern Pentathlon** |  | **11.509** |  | **3.064** |  | **3.756** |  | **0.793** |  | **0.005** | ****** |
| Artistics Gymnastics |  | Ice Hockey |  | 2.981 |  | 2.659 |  | 1.121 |  | 0.205 |  | 0.952 |  |
|  |  | Table Tennis |  | -5.319 |  | 3.266 |  | -1.629 |  | -0.367 |  | 0.733 |  |
|  |  | Trampoline |  | -4.954 |  | 3.159 |  | -1.568 |  | -0.341 |  | 0.769 |  |
|  |  | Rhythmic Gymnastics |  | -1.035 |  | 3.462 |  | -0.299 |  | -0.071 |  | 1.000 |  |
|  |  | Volleyball |  | -4.540 |  | 2.567 |  | -1.769 |  | -0.313 |  | 0.642 |  |
|  |  | Modern Pentathlon |  | -2.300 |  | 3.462 |  | -0.665 |  | -0.159 |  | 0.998 |  |
| **Ice Hockey** |  | **Table Tennis** |  | **-8.300** |  | **2.634** |  | **-3.151** |  | **-0.572** |  | **0.036** | ***** |
|  |  | **Trampoline** |  | **-7.935** |  | **2.500** |  | **-3.174** |  | **-0.547** |  | **0.034** | ***** |
|  |  | Rhythmic Gymnastics |  | -4.016 |  | 2.873 |  | -1.398 |  | -0.277 |  | 0.858 |  |
|  |  | **Volleyball** |  | **-7.521** |  | **1.692** |  | **-4.446** |  | **-0.518** |  | **< .001** | ******* |
|  |  | Modern Pentathlon |  | -5.281 |  | 2.873 |  | -1.838 |  | -0.364 |  | 0.594 |  |
| Table Tennis |  | Trampoline |  | 0.365 |  | 3.138 |  | 0.116 |  | 0.025 |  | 1.000 |  |
|  |  | Rhythmic Gymnastics |  | 4.284 |  | 3.442 |  | 1.245 |  | 0.295 |  | 0.918 |  |
|  |  | Volleyball |  | 0.779 |  | 2.541 |  | 0.307 |  | 0.054 |  | 1.000 |  |
|  |  | Modern Pentathlon |  | 3.019 |  | 3.442 |  | 0.877 |  | 0.208 |  | 0.988 |  |
| Trampoline |  | Rhythmic Gymnastics |  | 3.919 |  | 3.341 |  | 1.173 |  | 0.270 |  | 0.939 |  |
|  |  | Volleyball |  | 0.414 |  | 2.402 |  | 0.172 |  | 0.028 |  | 1.000 |  |
|  |  | Modern Pentathlon |  | 2.654 |  | 3.341 |  | 0.794 |  | 0.183 |  | 0.993 |  |
| Rhythmic Gymnastics |  | Volleyball |  | -3.506 |  | 2.788 |  | -1.257 |  | -0.242 |  | 0.914 |  |
|  |  | Modern Pentathlon |  | -1.266 |  | 3.628 |  | -0.349 |  | -0.087 |  | 1.000 |  |
| Volleyball |  | Modern Pentathlon |  | 2.240 |  | 2.788 |  | 0.803 |  | 0.154 |  | 0.993 |  |
|  | | | | | | | | | | | | | |
| * p < .05, ** p < .01, *** p < .001 | | | | | | | | | | | | | |
| *Note.*  P-value adjusted for comparing a family of 8 | | | | | | | | | | | | | |

**Table X2**

*Euclidean distances for cognitive functions separated by discipline*

| **Variable** | **Discipline** | **Mean (SD units)** | **Median (SD units)** | **Min (SD units)** | **Max (SD units)** | **N cases** |
| --- | --- | --- | --- | --- | --- | --- |
| processing speed | Basketball | 1.1 | 0.98 | 0 | 3.54 | 44 |
| processing speed | Ice Hockey | 1.21 | 0.98 | 0 | 4.32 | 50 |
| processing speed | Artistic Gymnastics | 0.77 | 0.69 | 0 | 2.55 | 23 |
| processing speed | Rhythmic Gymnastics | 0.64 | 0.49 | 0 | 2.26 | 19 |
| processing speed | Table Tennis | 0.95 | 0.83 | 0 | 2.75 | 21 |
| processing speed | Trampoline | 1.2 | 1.08 | 0 | 3.73 | 32 |
| processing speed | Volleyball | 1.1 | 0.98 | 0 | 3.83 | 98 |
| attention | Basketball | 1.11 | 0.89 | 0 | 3.56 | 11 |
| attention | Ice Hockey | 1.07 | 0.89 | 0.11 | 2.11 | 6 |
| attention | Artistic Gymnastics | 1.12 | 0.89 | 0 | 2.78 | 10 |
| attention | Rhythmic Gymnastics | 1.09 | 1 | 0 | 2.78 | 17 |
| attention | Trampoline | 1.22 | 1 | 0 | 4.79 | 22 |
| attention | Volleyball | 1.12 | 0.89 | 0 | 4.34 | 55 |
| working memory | Basketball | 1.17 | 0.88 | 0 | 5.26 | 54 |
| working memory | Ice Hockey | 1.02 | 0.88 | 0 | 4.38 | 50 |
| working memory | Artistic Gymnastics | 1.43 | 0.88 | 0 | 5.26 | 23 |
| working memory | Rhythmic Gymnastics | 0.81 | 0.88 | 0 | 3.5 | 19 |
| working memory | Table Tennis | 0.81 | 0.88 | 0 | 2.63 | 23 |
| working memory | Trampoline | 0.95 | 0.88 | 0 | 3.5 | 32 |
| working memory | Volleyball | 1.15 | 0.88 | 0 | 5.26 | 103 |
| cognitive inhibition | Basketball | 0.99 | 0.8 | 0 | 4.75 | 54 |
| cognitive inhibition | Ice Hockey | 1.36 | 1.13 | 0 | 6.15 | 50 |
| cognitive inhibition | Artistic Gymnastics | 1.08 | 0.88 | 0.01 | 3.92 | 23 |
| cognitive inhibition | Rhythmic Gymnastics | 0.99 | 0.77 | 0.01 | 3.86 | 19 |
| cognitive inhibition | Table Tennis | 1.04 | 0.85 | 0 | 3.13 | 23 |
| cognitive inhibition | Trampoline | 1.32 | 1.03 | 0 | 5.26 | 32 |
| cognitive inhibition | Volleyball | 1.01 | 0.86 | 0 | 5.01 | 103 |
| cognitive flexibility | Basketball | 1.07 | 0.91 | 0 | 3.97 | 54 |
| cognitive flexibility | Ice Hockey | 0.99 | 0.81 | 0 | 4.69 | 50 |
| cognitive flexibility | Artistic Gymnastics | 1.29 | 1.13 | 0.01 | 3.82 | 23 |
| cognitive flexibility | Rhythmic Gymnastics | 1.41 | 1.09 | 0.01 | 5.92 | 19 |
| cognitive flexibility | Table Tennis | 1.39 | 1.02 | 0.01 | 6.59 | 23 |
| cognitive flexibility | Trampoline | 0.88 | 0.78 | 0 | 2.98 | 32 |
| cognitive flexibility | Volleyball | 1.01 | 0.8 | 0 | 6.51 | 103 |
